# The Small Size and Superficial Location Suggest That Laparoscopic Partial Nephrectomy Is the First Choice for the Treatment of Juxtaglomerular Cell Tumors

**DOI:** 10.3389/fendo.2021.646649

**Published:** 2021-04-30

**Authors:** Zixing Ye, Hua Fan, Anli Tong, Yu Xiao, Yushi Zhang

**Affiliations:** ^1^ Department of Urology, Peking Union Medical College Hospital, Beijing, China; ^2^ Department of Endocrinology, Peking Union Medical College Hospital, Beijing, China; ^3^ Department of Pathology, Peking Union Medical College Hospital, Beijing, China

**Keywords:** juxtaglomerular cell tumors, small size, superficial location, laparoscopic partial nephrectomy, laparoscopic ultrasound

## Abstract

**Background:**

Juxtaglomerular cell tumor (JGCT) is a very rare disease, and surgical resection is the only possible way to cure this tumor. Open nephrectomy and partial nephrectomy have been reported to manage JGCTs with excellent results in the previous reviews. Laparoscopic surgery has been popularized in recent years, while critical issues associated with laparoscopic surgical management have been seldom reported. We summarized the JGCTs in our center to discover the optimal surgical management and its anatomic foundation.

**Methods:**

In this retrospective study, we enrolled a total of 14 JGCT patients. All patients received surgeries and were followed up for up to 11 years. We mainly summarized the size and location of tumors, imaging features, and surgical strategies. A descriptive statistical analysis was performed.

**Results:**

The JGCTs in this study had a median size of 1.35 cm and all located superficially, mainly in the cortical or subcortical area of the kidney. All 14 patients had hypertension, ten had hypokalemia, and seven had elevated plasma renin activity. Pathologically, JGCT cells were polygonal or spindle shape, with positive CD34 and vimentin immunostaining. All patients received partial nephrectomy; nine were laparoscopic, and five were open. Laparoscopic partial nephrectomy (LPN) was performed in seven out of eight patients over the last nine years. Postoperative blood pressure, serum potassium, and plasma renin activity were normal in all patients. No recurrence occurred within a median follow-up of 60 months.

**Conclusion:**

The small size and superficial location are the characteristic anatomic features of JGCT; they suggest that LPN is the preferred surgical strategy. Laparoscopic ultrasound is helpful for the intraoperative detection of small JGCTs. Longer follow-up is required to examine the biological behavior of JGCTs and the effect of LPN.

## Introduction

Juxtaglomerular cell tumors (JGCTs) of the kidney are rare renin-producing tumors, first described by Robertson in 1967 (1). Over the past 50 years, an estimated 100 cases have been reported worldwide (2). Hypertension and hypokalemia, the secondary symptoms of excessive renin secretion, are the most common presentations. Despite the benign nature of most JGCTs, long-term hypertension from a young age can cause end-organ damage. Current literatures have mostly focused on the diagnosis of JGCTs. Surgical management, the only possible way to cure this tumor, has yet to be fully addressed, mostly due to a lack of cases and limited surgical experience (3–5). Previous literature review in 2008 summarized most JGCT cases at that time and showed that open nephrectomy and partial nephrectomy could manage JGCTs excellently, while only one laparoscopic partial nephrectomy (LPN) was mentioned then (6). In recent years, LPN has become popular for renal tumors, and many case reports have shown the feasibility of LPN for JGCTs. In this study, we reported 14 JGCT cases who underwent complete tumor resection in our institution, representing the largest single-center study of JGCTs thus far. We suggested that small size and superficial location are the characteristic anatomic features of JGCTs, which determined the surgical strategy.

## Patients and Methods

### Patients

We retrospectively studied 14 patients who were pathologically diagnosed with JGCT in our institution between January 2005 and October 2019. All patients, except two, were clinically diagnosed with JGCT preoperatively, based on typical manifestations, endocrinological examinations, and imaging examinations, including hypertension, hypokalemia, increased plasma renin activity (PRA), and the presence of a kidney tumor. All patients received partial nephrectomy with an open or laparoscopic approach. Nine patients were followed-up after surgery, ranging from three months to 11 years. The other five patients were lost to follow-up after discharge.

### Methods

#### Data Collection

Demographic parameters, medical history, clinical manifestations, blood pressure, serum potassium concentration, plasma aldosterone concentration (PAC), and PRA were examined preoperatively. Conventional ultrasonography, non-contrast computed tomography (NCCT), and contrast CT were performed in all patients. Contrast magnetic resonance imaging (MRI) and contrast ultrasonography were performed in three patients whose NCCT did not show clear images. Blood pressure, serum potassium, PRA, and PAC were assessed postoperatively. PRA and PAC were examined in a standing position. The first follow-up began in the third month after surgery. Pathological examinations, including histology and immunostaining, were analyzed routinely.

#### Surgical Strategy

LPN was performed preferentially. Tumors were resected completely, similar to renal cell carcinoma. Operation time, renal artery blocking time, surgical complications, and blood transfusion were recorded.

#### Evaluation of Endocrinological Examinations

The cut-off values for elevated PRA and elevated PAC were 6.56 ng/mL/h and 23.9 ng/dL in a standing position, which were the upper limits of the normal range in our institution.

#### Statistical Analyses

This study was a descriptive study. Median values and interquartile ranges (IQRs) were used to describe all continuous variables. Proportions and frequencies were used to describe categorical variables. Statistical analysis was performed using SPSS software, version 23.0 (SPSS Inc., www.spss.com).

### Ethics Approval

The study was approved by the Institutional Review Board of Peking Union Medical College Hospital. The data are anonymous, and the requirement for informed consent was therefore waived.

## Results

### Anatomic Features

Most of the JGCTs were small and superficial. The detailed location and size of the JGCTs were summarized in **Table 1**. The median tumor diameter was 1.35cm (IQR: 1.10–2.38 cm), ranged from 0.7 - 4.8 cm. Only four tumors were more than 2.0 cm in diameter; these were all diagnosed nine years ago or even earlier. Two tumors were completely invisible during surgery. Three tumors were located in the upper pole of the kidney, six were in the middle, and five were in the lower pole.

**Table 1:** Detailed information for all JGCT patients.

Case No.	Basic information		Tumor size (cm)	Imaging examinations					Surgery			Pre-operative examinations			Follow up			
	Sex/Age of diagnosis (year-old)	Course of disease		NCCT		Contrasted CT CT value (HU)		Ultrasound features	Strategy	Operation time (min)	Renal artery blocking time (min)	serum potassium (mmol/L)	PRA (ng/ml/h)	BP (mmHg)	Follow -up period (month)	serum potassium (mmol/L)	PRA (ng/ml/h)	BP (mmHg)
				visibility	CT value (HU)	Cortical phase	Parenchymal phase											
1	M/16	2y	2.5	Y	37	61	NA	hypo	LPN	105	28	3.0	>12	220/140	132	4.7	4.83	125/85
2	M/46	6y	2.0	Y	40	NA	NA	iso and hyper	OPN	90	15	3.5	NA	125/80*	–	4.3	NA	110/75
3	F/37	7y	4.8	Y	29	104	NA	hypo	LPN	160	58	2.3	>12	200/140	3	3.6	1.26	135/85
4	F/18	1m	4.2	Y	35	NA	NA	hypo	OPN	60	25	3.8	>12	240/130	–	3.8	4.5	130/80
5	M/40	12y	1.5	N	47	75	NA	iso	LPN	90	24	3.8	NA	140/95*	84	4.1	0.67	132/94
6	F/29	1m	0.7	N	35	55	120	invisible	LPN	90	16	2.5	6.17	180/145	84	4.9	1.66	130/95
7	M/10	2y	1.2	Y	40	70	NA	invisible	LPN	110	17	2.8	>12	209/180	–	4	NA	117/71
8	F/34	4y	1.1	N	34	104	107	hypo	LPN	60	13	3.0	>12	170/100	3	4.5	1.86	102/64
9	F/29	1y	1.2	N	30	66	124	iso	LPN	60	15	2.8	>12	220/180	6	4.1	0.65	130/80
10	M/39	11y	1.1	Y	NA	NA	NA	hypo	LPN	50	0	2.7	18	180/120	108	4.2	1.47	120/80
11	F/26	4m	1.8	N	NA	NA	NA	Iso and hyper	OPN	90	18	3.9	0.3	200/110	–	4.8	NA	110/70
12	F/15	1y	1.0	N	NA	NA	NA	invisible	OPN	75	0	2.5	3.5	245/135	–	4.5	0.3	130/80
13	F/72	3y	2.9	Y	38	92	105	hypo	OPN	100	20	3.8	NA	130/80*	60	4.5	NA	125/60
14	F/28	4y	0.7	N	33	53	81	iso and hyper	LPN	45	10	3.1	4.36	200/120	3	3.9	1.55	113/89

M, male; F, female; m, month; y, year; R, right; L, left; Y, yes; N, no; iso, isoechoic; hypo, hypoechoic; hyper, hyperechoic; LPN, laparoscopic partial nephrectomy; OPN, open partial nephrectomy; PRA, plasma renin activity; PAC, plasma aldosterone concentration; NA, not available; BP, blood pressure.

^*^Blood pressure was measured when anti-hypertension medications were used, and the maximum BP was unknown.

**Figure 1:**
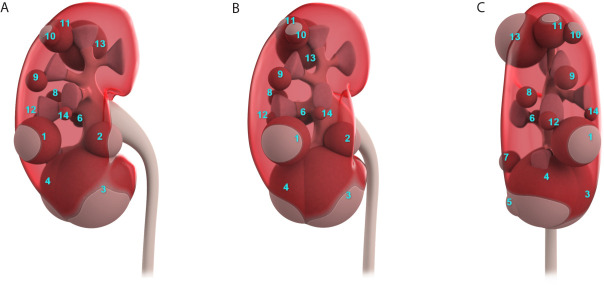
The actual location and size of all 14 juxtaglomerular cell tumors were summarized in one schematic diagram. **(A–C)** show the schematic diagram’s views at 0°, 30°, and 90°.

The length, width, and thickness, of the model kidney (**Figure 1**) were 11.5 cm, 5.9 cm, and 4.8 cm, respectively, based on human data from previous studies (7). The location and size of the JGCTs were determined by CT or MRI examinations. Views of the schematic diagram at 0°, 30°, and 90° are shown in **Figures 1A–C**. As shown, most tumors were small, superficial, and mainly within the cortex. There is no obvious preference for the location of the tumors in the kidney.

### Clinical Features of Patients

Fourteen cases of JGCT were included: five males and nine females (**Table 1**). The median age of onset of symptoms was 28 years (IQR: 19.5 - 29.75 years), and 12/14 patients were under 30 years old at the time of diagnosis. The median course of the disease was 30 months (IQR: 12-66 months). During the disease, all 14 patients suffered from hypertension, and ten had hypokalemia. In a standing position, seven patients had elevated PRA, and four patients had elevated PAC.

### Imaging Examinations

**Figure 2:**
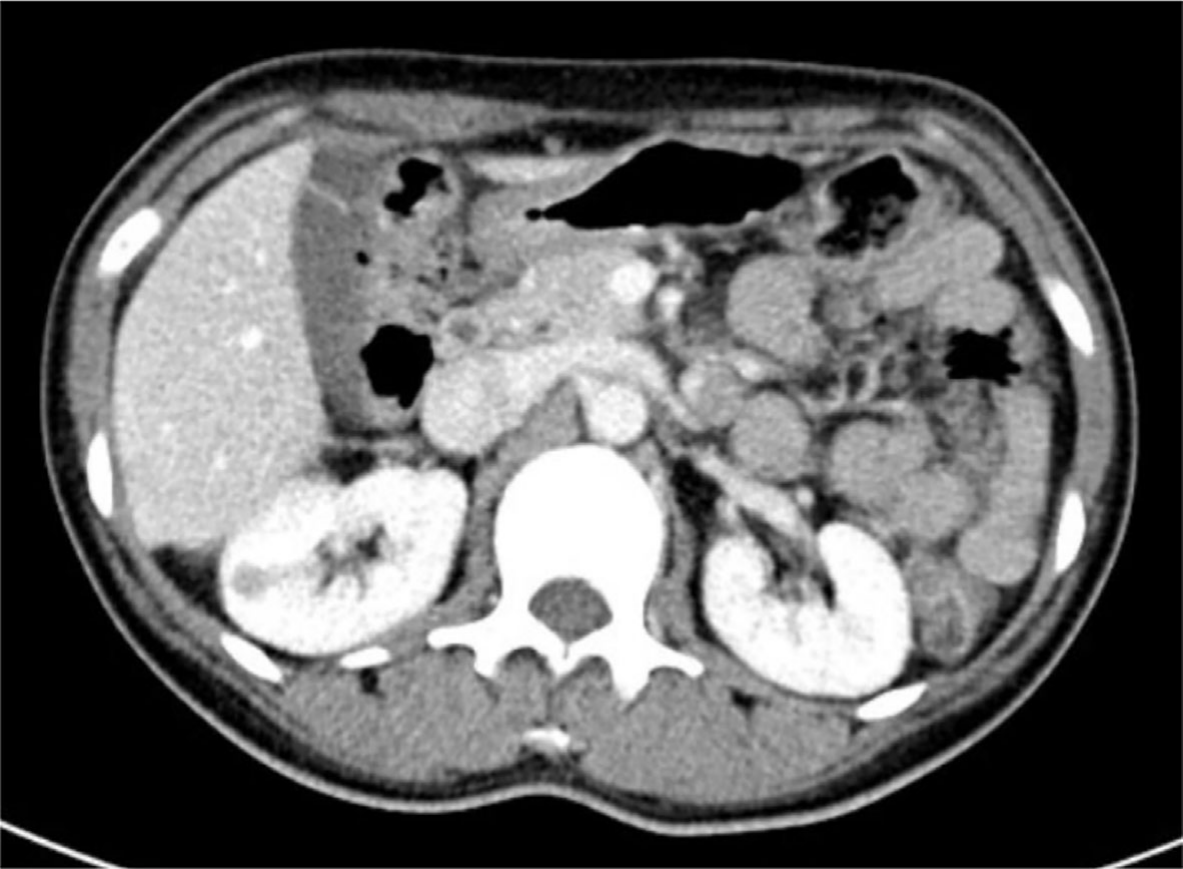
The parenchymal phase of contrast computed tomography image of Patient No. 8.

Contrast CT distinguished all 14 JGCTs; NCCT recognized only half of the tumors, and only when the tumors protruded obviously from the kidney. All JGCTs showed isodensity in the non-contrast phase, with a median CT value of 35 HU (IQR: 33.5-39 HU). The median CT value was 70 HU (IQR: 61–92 HU) and 107 HU (IQR: 105–120 HU) in the cortical and parenchymal phase, respectively; both of these values were lower than the surrounding kidney cortex. In the cortical phase, the JGCT located beneath the cortex may be indistinguishable from the less-enhanced medulla. The tumor was the clearest in parenchymal phase or early excretory phase, because the kidney was generally enhanced in this phase, but the tumor was still less-enhanced. (**Figure 2**).

Conventional ultrasonography was performed 29 times in 14 patients, and false-negative results occurred in nine of the 14 patients. The smallest tumor detected by ultrasound was as small as 1.1 cm, and the largest tumor missed by ultrasound was 2.5 cm in diameter. Six tumors were hypoechoic, five were mainly isoechoic, and three were invisible by conventional ultrasonography. In contrast ultrasonography, the tumor showed the same enhancement levels as the cortex in the arterial phase; subsequently, the level of enhancement diminished quickly, and the tumor became less enhanced than the cortex in the venous phase.

**Figure 3:**
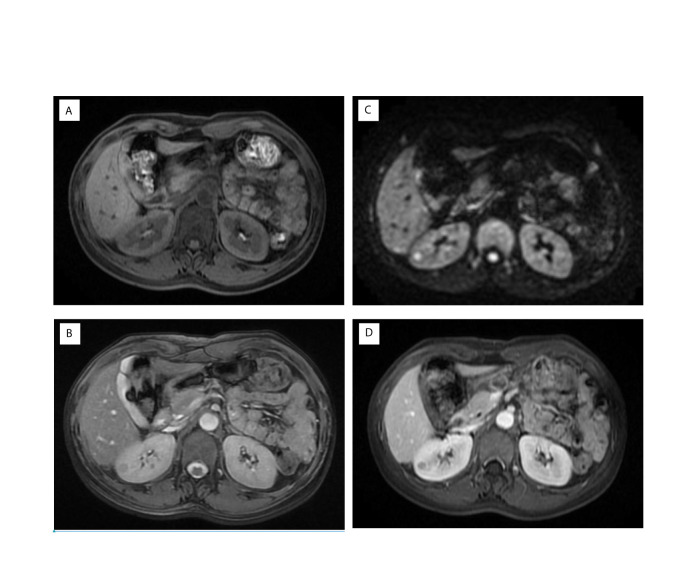
Magnetic resonance imaging (MRI) images of Patient No. 8. Signals of **(A)** T1, **(B)** T2, and **(C)** Diffusion-weighted images of non-contrast MRI. Signal of **(D)** arterial phase of contrast MRI.

In non-contrast MRI, all three JGCTs were isointense on T1-weighted images (WIs) and hyperintense on diffusion-WIs (**Figures 3A, C**). Two showed hyperintense on T2-WIs, and one showed a hypointense on T2-WIs (**Figure 3B**). In contrast MRI, all JGCTs showed less enhancement than the cortex in the arterial phase (**Figure 3D**).

### Surgical Strategies

**Figure 4:**
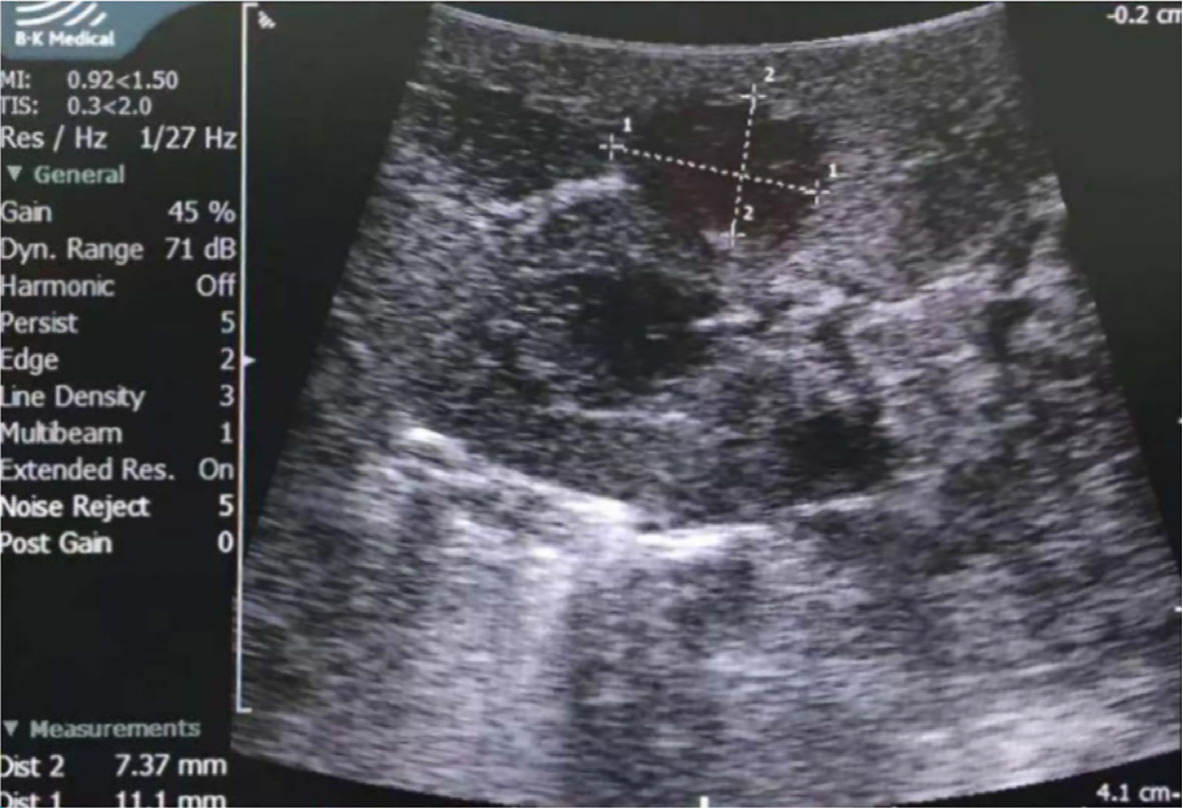
Laparoscopic ultrasound of Patient No. 8. The tumor was hypoechoic and was measured 7.4×11.1mm by ultrasound.

All patients received partial nephrectomy; nine were laparoscopic, and five were open. LPN was performed in seven out of eight patients over the last nine years. Hypothermic renal artery perfusion *in situ* was performed in Patient No. 3 because the patient enrolled in a clinical research project; this significantly prolonged the operation time and renal artery blocking time. Apart from this patient, the median operation time was 90 min (IQR: 75–90 min) and 90 min (IQR: 60-105 min) for open and laparoscopic surgery, respectively; the renal artery blocking time was 18 min (IQR 15-20 min) and 16 min (IQR 13–24 min), respectively. All five open surgeries were performed before 2011 (since 2005), with a median operation time of 90 min (IQR: 75-90 min) and a renal artery blocking time of 20 min (IQR: 15-24 min). Three laparoscopic surgeries were performed in the recent three years, with a median operation time of 60 min (IQR: 52.5-60 min) and a renal artery blocking time of 13 min (IQR: 11.5-14 min). Although the open PN (OPN) and LPN were not performed in similar years, both were performed by skilled surgeons. Laparoscopic ultrasound was applied in four patients, whose tumors could not be distinguished in NCCT; two tumors were completely invisible during surgery, and the other two protruded from the kidney slightly (**Figure 4**). Neither blood transfusion nor complication occurred.

### Pathological Features

**Figure 5:**
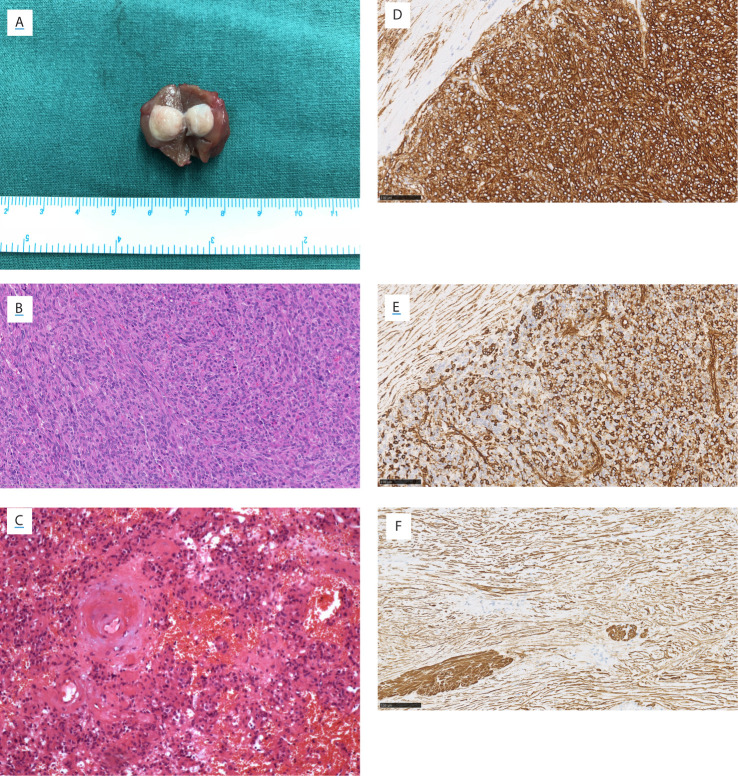
Gross view, microscopic view, and immunostainings of Patient No. 8. **(A)** Gross view. Microscopic view (HE staining) showing **(B)** the polygonal and spindle cells, **(C)** the thickened hyalinized vessel wall and focal hemorrhage. The immunostaining of **(D)** CD34, **(E)** vimentin, and **(F)** smooth muscle actin. **(B–F)** Magnification ×200.

All diagnoses were made by pathological examinations. Patients No.2 and No.13 were diagnosed with JGCT accidentally. Tumors were encapsulated and well-circumscribed, without the involvement of the renal sinus or perinephric fat (**Figure 5A**). Microscopic examination revealed that the tumors were composed of polygonal and spindle cells (**Figure 5B**), the mitosis was rare. There were plenty of blood sinuses in the parenchymal tissue. Blood vessels within the tumor were significantly hyalinized, and hemorrhage was focally present in a few cases (**Figure 5C**) (4). Ki-67 immunostaining was performed in 13 patients. A Ki-67 index >2% was observed in three tumors (3%, 6%, and 10%).

Immunostaining is useful in the diagnosis and differential diagnosis of JGCT. Thirteen patients performed the CD34 and Vimentin staining, and all were positive (**Figures 5D, E**). Vascular endothelial growth factor (VEGF) was positive in 11 patients who underwent this staining. Smooth muscle actin (SMA) was positive in nine of 14 patients (**Figure 5F**). Periodic acid schiff stain was positive in six of seven patients. Immunostainings of CgA, Human melanoma black 45 (HMB45), S100, AE1/AE3, and Desmin were performed in most of 14 patients, respectively; and all these stainings were negative.

### Follow-Up

Nine patients were followed-up from three months to 11 years, with a median follow-up of 60 months (IQR: 3–84 months). In all these patients, symptoms, serum potassium, blood pressure, and PRA, were normal within three months after the surgery. No recurrence was observed in their latest follow-up. Five patients were lost after discharge.

## Discussion

In this study, all the JGCT patients in the recent nine years received LPN, and all the symptoms, blood pressure, serum potassium, and PRA were back to normal within three months after the surgery. We suggested that the small size and superficial location of tumors guaranteed the effectiveness of LPN.

### Small Size of JGCT and the Pathological Foundation

Poor blood supply was an important reason underlying the small size of JGCTs. Arteriography in the previous studies showed that JGCTs were hypovascular with only minimal neovascularity (8). Although pathological examinations revealed plenty of vessels in parenchymal tissue and high expression levels of angiogenesis biomarkers, including CD34 and VEGF; blood vessels inside the tumor were either small and thin-walled, or had a significantly thickened and hyalinized wall (4, 9). It suggested that abnormalities in the vessel wall, rather than insufficient angiogenesis, ultimately lead to the JGCT’s poor blood supply. The exact reason for abnormality in the vessel walls remains unknown, but probably relates to the excessive secretion of renin (10). Rare mitotic activity and low or absence of Ki-67 expression level indicated that the JGCTs were not actively proliferating; this could explain their small size.

The wider recognition of JGCT is another crucial reason for the small size of JGCT. The mean diameter of JGCTs reported in global studies in 1983, 1993, and 2019 decreased from 4.1 cm to 2.9 cm, and then to 1.8 cm (5, 8, 11). Over the past seven years, all six JGCTs diagnosed in our institution were less than 1.5 cm; before this study, the mean diameter of JGCTs was reported to be 2.5 cm. Severe hypertension is uncommon at a young age, as is hypokalemia. These urge physicians to carry out detailed examinations and always led to the identification of JGCT.

### Superficial Location of JGCT and the Pathological Foundation

Superficial location was another feature of JGCT. Most of the previous case reports have shown that JGCTs located in the cortical or corticomedullary location (5, 12, 13). However, unlike the renal cell carcinoma, some JGCTs, which were as large as more than 3 cm, could be endophytic entirely in kidney (14–16). Juxtaglomerular cells are a modified form of the smooth muscle cell, formed from a vascular component of the juxtaglomerular apparatus, and mainly located in the walls of the afferent arterioles (17). JGCT thus could originate from the cortex and renal column of the kidney. These JGCTs that were completely endophytic probably originated from the renal column’s juxtaglomerular apparatus. Because there are more glomeruli in the cortex than in the renal column, most JGCTs were superficial.

### Multiple Imaging Modalities in the Diagnosis of JGCT

JGCTs were always small and endophytic. In this study, more than half of these tumors were missed by ultrasound or NCCT. Multiple imaging modalities might be necessary for the diagnosis of JGCTs.

Contrast CT is the most commonly used and effective imaging modality in the diagnosis of JGCT at present and can show hypovascular tumors in cortical, parenchymal, and excretory phases (3, 13). However, the small JGCTs could still be misdiagnosed as kidney cysts by contrast CT (6). In our recent cases, JGCTs were always small and confined inside the kidney; this made multiple imaging examinations important.

MRI has recently been recognized as an effective and sensitive imaging examination, especially when tumors are not evident in contrast CT. The diffusion-WIs are related to the motion of water molecules and are highly sensitive for small lesions. All three JGCTs, including tumors as small as 0.7 cm × 0.7 cm, were significantly hyperintense on diffusion-WIs, as described previously (5, 18). Unlike contrast CT, MRI provides more modalities, without enhancement, and does not affect the kidney function. MRI, therefore, represents a good alternative for contrast CT. Clear cell renal cell carcinoma (ccRCC) is the most common renal cancer. Although ccRCC has typical avid enhancement in the cortical phase of contrast CT, it is quite difficult to judge whether a renal tumor is enhanced or not when it is small due to volume-averaging effect. MRIs of ccRCC show low signals in T1-WIs and were hypointense on diffusion-WIs (19). Papillary RCC was hypovascular; the enhancement level increased gradually and peaked during the parenchymal phase in contrast CT. However, papillary RCC showed hypointense signals in diffusion-WIs (19). MRI was, therefore important in differentiating JGCTs from other small or hypovascular renal tumors.

Contrast ultrasound also detected small JGCTs in our study, as MRI did, and represents a potential imaging modality for JGCT examination; however, very few studies have mentioned this previously (15). It needs more experience to confirm the efficacy.

The largest tumors missed by NCCT and conventional ultrasound were 1.8 cm and 2.5 cm, respectively; this suggests a false-negative rate as high as 64.2% and 78.6%, which could be even higher since tumors are getting smaller. Thus, NCCT and conventional ultrasound are not suggested for JGCT screening.

### Pathological Features and the Diagnosis of JGCTs

Although most JGCTs were clinically diagnosed through manifestations, pathological features were crucial in the final diagnosis. The histological features of our cases were consistent with previous studies (9). Immunostaining was essential for the diagnosis as well. The markers representing the vessel origin were characterized in JGCT, including CD 34, Vimentin, and VEGF. SMA was also positive in most cases, shown in previous studies (17). The immunostaining of renin and detection of renin granules on ultrastructural studies were used to confirm the diagnosis, although the renin could also exist in Wilms tumor, RCC, and renal oncocytoma (20). The renin staining was not shown in our cases since it was not routinely performed in our institution, and it should be performed in complex cases.

Several renal tumors should be considered in the differential diagnosis, including hemangiopericytoma, RCC, and metanephric adenoma. JGCT was classified as hemangiopericytoma before, and had a hemangiopericytoma-like growth pattern; however, current studies found that hemangiopericytoma did not have the thick-walled vessels and polygonal cells and was CD34 and SMA negative (17). JGCT with papillary growth pattern may resemble papillary RCC, while RCC lacks the polygonal and spindle cells. Metaneprhic adenoma was a rare benign stromal cell tumor that may resemble the JGCT, while it has the characteristic psammoma body.

Using comparative genomic hybridization and interphase fluorescence in situ hybridization, monosomy of Chromosome 9, 11, 15, 21 and X, and polysomy of Chromosome 10 and 18 have been found in JGCT (9, 21). Several up-regulated genes were found in Chromosome 4 and 10. Down-regulated genes were found in Chromosome 9, 20, and X (9). The functions of these genes need to be further investigated.

Although malignant JGCT was rarely reported, recurrence, metastasis, and vessel invasion were found in several cases (22). In our cases, three cases have Ki-67 more than 2%, which require a longer follow-up.

### Laparoscopic Partial Nephrectomy Is the Preferred Surgical Strategy

A previous review in 2008 summarized 89 cases; only one of these received LPN (6). Because JGCT can be found when it is smaller and more superficial, and the surgical technique of LPN has been greatly improved over the recent years, all patients over the last nine years received LPN in our study. For all patients, there are similar operation time, renal artery blocking time, and safety between laparoscopic and open surgery, which indicates that LPN is safe and effective for JGCTs (6). Unfortunately, it is now difficult to compare LPN and OPN because there are very few JGCT cases requiring OPN. However, we believe that laparoscopic surgery will become the preferred surgical method, with the advancement of laparoscopic surgery, especially the development of robotic surgery.

Although malignant JGCTs were rarely reported, one of our JGCTs had the Ki-67 index as high as 10%; we thus suggested that JGCTs should be resected using the same range as RCC because of the lack of long-term follow-up data (22).

Because of the small size of JGCTs and the popularity of laparoscopic surgery, there is a significantly increased risk of missing JGCTs during surgery. The laparoscopic ultrasound has been applied in previous cases when JGCTs were entire endophytic (14, 15). In this study, four JGCTs that were endophytic and indistinguishable in NCCT received the laparoscopic ultrasound. Laparoscopic ultrasound is thus crucial to help localize the JGCTs, delineate the border, and help differentiate JGCTs from other nearby renal masses when JGCTs are endophytic in preoperative NCCT and when there is more than one tumor in the same kidney.

### Limitations

There were limitations to this study. First, this was a single-center retrospective study. Also, five patients were lost after discharge, which made it difficult to assess the recurrence of JGCTs.

## Conclusions

The small size and superficial location are the characteristic anatomic features of JGCT, which determines that multiple imaging modalities are required to diagnose the JGCT and LPN is the preferred surgical strategy. Laparoscopic ultrasound is particularly helpful for detecting small JGCTs and determining the tumor border during surgery. Longer follow-up is needed to examine the biological behavior of JGCTs and the effect of LPN.

## Data Availability Statement

The original contributions presented in the study are included in the article/supplementary material. Further inquiries can be directed to the corresponding author.

## Ethics Statement

The studies involving human participants were reviewed and approved by Institutional Review Board of Peking Union Medical College Hospital. Written informed consent to participate in this study was provided by the participants’ legal guardian/next of kin.

## Author Contributions

ZY: research idea and study design, data acquisition, data analysis/interpretation, and supervision or mentorship. HF: statistical analysis and data analysis/interpretation. AT: data acquisition and supervision or mentorship. YX: data acquisition. YZ: research idea and study design, data analysis/interpretation, and supervision or mentorship. Each author contributed important intellectual content during manuscript drafting or revision, accepts personal accountability for the author’s own contributions, and agrees to ensure that questions pertaining to the accuracy or integrity of any portion of the work are appropriately investigated and resolved. All authors contributed to the article and approved the submitted version.

## Conflict of Interest

The authors declare that the research was conducted in the absence of any commercial or financial relationships that could be construed as a potential conflict of interest.
